# Why (and how) we should publish negative data

**DOI:** 10.15252/embr.201949775

**Published:** 2019-12-20

**Authors:** Simon Nimpf, David A Keays

**Affiliations:** ^1^ Research Institute of Molecular Pathology (IMP) Vienna Biocenter (VBC) Vienna Austria

**Keywords:** Methods & Resources, S&S: Ethics, S&S: Economics & Business

## Abstract

Negative data and refutations are a crucial element of the scientific process. But it needs solid arguments to convince editors and reviewers to publish negative results.

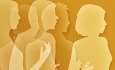

Central to the scientific method are the concepts of falsifiability and hypothesis testing: We cannot prove a hypothesis, merely acquire evidence that supports or refutes it 1. We should reject those ideas that we can refute and advance those that we cannot. In this respect, the generation and publication of negative data lie at the very heart of the scientific enterprise, and yet, there is an overwhelming focus on positive findings. Whether it is a PhD student, postdoc, lab head or an editor, there is a great reluctance to publish a paper that begins with the word “No”. In fact, the percentage of papers declaring support for a tested hypothesis has increased by 22% between 1990 and 2007 across scientific disciplines 2. This has served to fuel the exaggeration and distortion of scientific findings, which has led to the “reproducibility crisis”. Current evidence suggests that between 51 and 89% of published studies cannot be reproduced 3, 4, 5, a fact which has even caught the attention of the mainstream media. It has considerable economic consequences, resulting in some $28 billion of wasteful spending per year in the USA alone 6. Failure to publish negative data ensures that dubious ideas and wrong‐headed projects receive financial support with multiple groups toiling away in vain, when that money and time could be spent on more productive endeavors 7. So, the philosophical, practical, and economic arguments for publishing negative data are strong—but what is the best way to do this?

Let us assume that you are an idealistic PhD student who has spent the better part of three years attempting to replicate a much‐hyped finding that was originally published in a top‐tier journal. Much to your dismay, and despite your very best efforts, a successful replication has eluded you. How should you proceed? First, it is important to appreciate that failure to reproduce a study can arise for a number of reasons. While the initial hypothesis may be false, it may also be attributed to different reagents or conditions, lack of statistical power, or the complexity of the biological system. For this reason, a replication study should use the exact same reagents and methods, and in the case of cellular experiments, the identical cell line. The latter is particularly important, as it has been shown that cell lines are not genetically stable and that lines from different labs are functionally distinct. Whole genome analysis of 27 strains of the MCF2 breast cancer cell line showed that ten chromosome arms were differentially gained or lost, and more than 688 copy number variations were detected. This genetic heterogeneity had a major impact on the viability of these cells when exposed to a panel of 321 chemotherapeutic drugs 8. Moreover, even when different labs have been provided with the exact same clone (MCF10A) and a common set of reagents, large differences (> 200 fold) in the cells’ sensitivity to anti‐cancer drugs have been reported 9. This has been attributed to variation in cell counting, compound handling, and pipetting 9. It is evident that even small departures from an established protocol can have a profound effect on the results of an experiment. For this reason, it is prudent to first contact the authors of the original study and seek their advice as to whether any technical issues might be the underlying problem for the failure to reproduce their findings. A visit to their laboratory might likewise prove to be a valuable exercise to resolve the issue.

Nevertheless, in some instances the problem lies with a primary hypothesis that is false and publication of your data will be imperative. What are your options? A number of scientific publishing houses established journals such as *New Negatives in Plant Science* (Elsevier) or the *Journal of Negative Results in BioMedicine* (Springer) to specifically publish negative results and refutations, but both have now been discontinued. Only the *Journal of Negative Results* (which published a single article in 2018) and the *Journal of Articles in Support of the Null Hypothesis* remain.

These avenues are not very attractive to students or postdocs who need a fellowship to continue their scientific careers. For this reason, we encourage authors to submit negative data to the journal in which the original manuscript was published. Editors may be little reluctant to accept manuscripts that question high‐profile findings, but they are open to the persuasive argument that hypothesis testing requires both refutation and replication in equal measure. While railing against editors might be a favorite pastime of scientists, most editors are conscience of their privileged position to ensure the integrity of peer review and the scientific record. In fact, many reputable journals have clear policies that detail how to handle and accept refutations.

If a journal chooses to send your refutation out for review, it will likely give the original authors an opportunity to respond to your manuscript. In this position, many scientists will defend their original findings and will attempt to cast doubt on their challengers by focusing on methodological discrepancies. Accordingly, to convince editors and reviewers, you should emphasize the lengths that you went to adopt the same protocols and use the same reagents. It is advisable to assume a measured and neutral tone, and to offer a number of reasons that might explain your failure to replicate this particular study. Your goal should be to persuade the editors and the scientific community that your study is superior. This objective will be advanced by demonstrating lack of reproducibility in another system, or through the addition of orthogonal experiments.

Submitting your manuscript to the journal in which the original paper appeared will be successful sometimes, but not always. In this case, we advocate submission to an open‐access journal, such as *PLoS One*, that accepts technically sound manuscripts without specific requirements for novelty. Alternatively, pre‐print servers, such as the BioRxiv platform, enable authors to publish their data swiftly in a citable form with the potential to ignite interest and attention. Overall, the responsibility lies with us, the scientific community, to recognize the importance of negative data and to make sure it is made public.
